# Supplementary material for: Fungal Hyphosphere Microbiomes Are Distinct from Surrounding Substrates and Show Consistent Association Patterns
Source: Microbiol Spectr. 2023 Mar 20;11(2):e04708-22. doi: 10.1128/spectrum.04708-22 (PMC10100729; doi:10.1128/spectrum.04708-22)
Supplement: Supplemental file 1 — Fig. S1 to S5 and Table S1. Download spectrum.04708-22-s0001.pdf, PDF file, 1.1 MB [file spectrum.04708-22-s0001.pdf]

Supplementary Figures and Tables

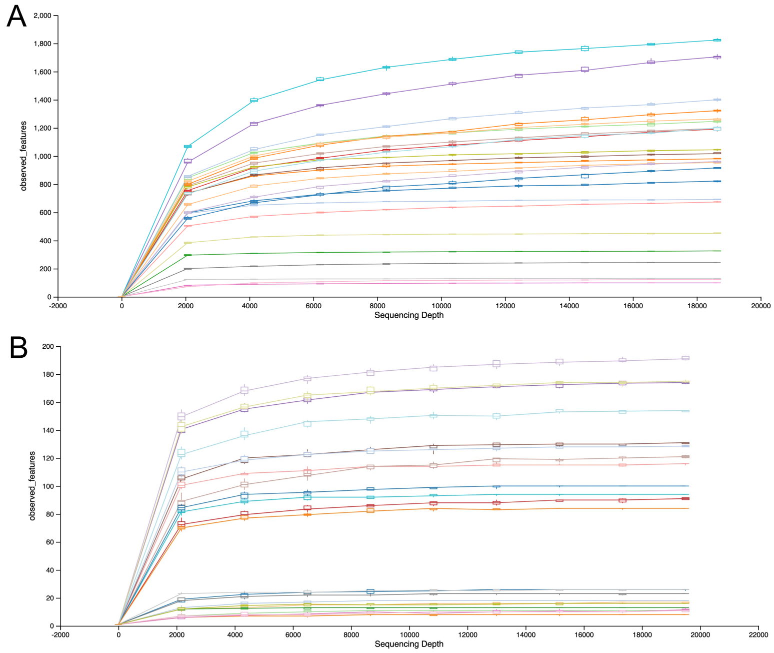

Supplementary Figure 1. Rarefaction of A) bacterial and B) fungal datasets used in this manuscript showing that most of the samples have either reached or getting close to saturation. Observed features (ASVs) within mats are saturated whereas some samples outside of the mats are getting close to saturation. The bacterial dataset was rarefied at 18640 sequences and the fungal dataset was rarefied at 19500 sequences.

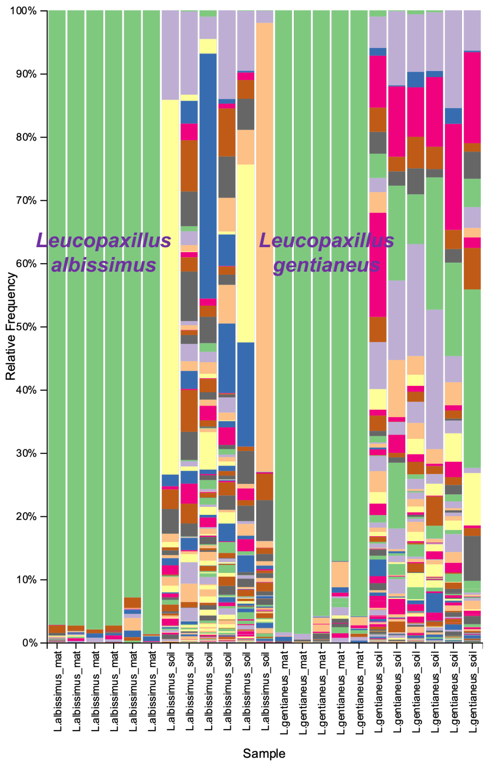

Supplementary Figure 2. Stacked-barplot of the 100% relative abundance of the fungal taxa that occurred within fungal mat and non-mat samples. *Leucopaxillus albissimus* and *L. gentianeus* dominated the fungal communities within their respective mats.

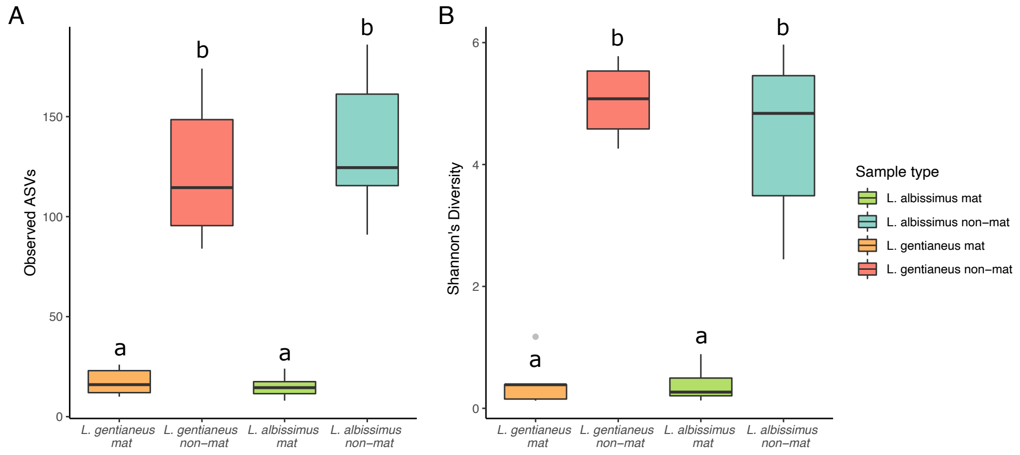

Supplementary Figure 3. Fungal A) observed richness and B) Shannon's Diversity compared among mat and non-mat samples across the two fungal host species. Observed richness and Shannon's Diversity were significantly different between mat and non-mat samples ($p < 0.007$). Mat samples were not significantly different from each other. Non-mat samples were not significantly different from each other.

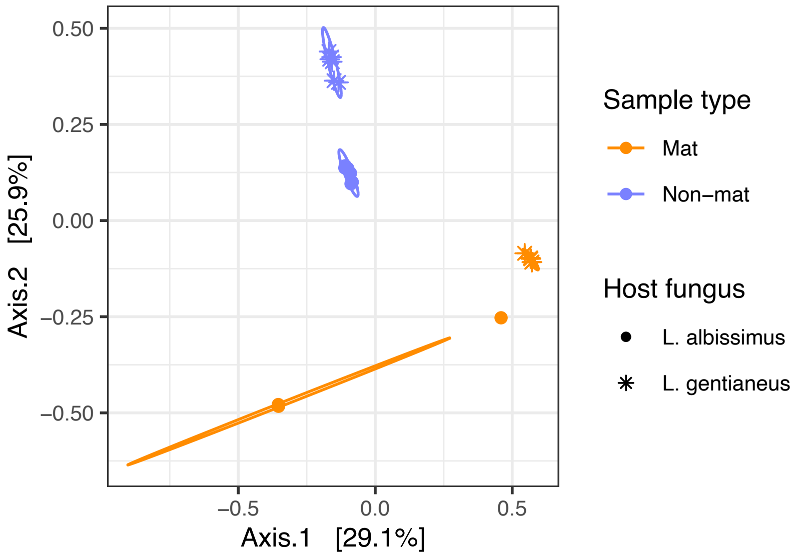

Supplementary Figure 4. PCoA ordination of the fungal community in mat and non-mat samples across the two different *Leucopaxillus* species. The ellipses are drawn to group sample types and fungal host species. Bray-Curtis distance was used in this ordination.

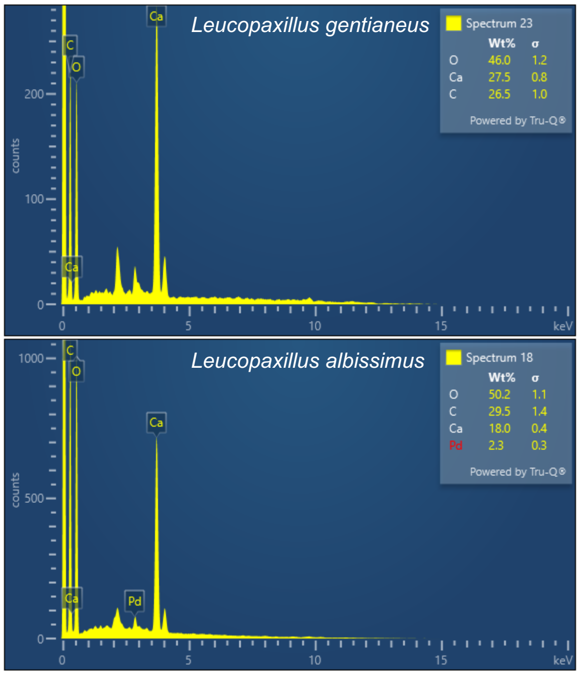

Supplementary Figure 5. Energy-Dispersive X-ray Spectroscopy (EDS) signatures of the crystalline structures formed outside of *Leucopaxillus* hyphae. The makeup and proportion of the individual elements correspond well to the elemental makeup of calcium oxalate.

Comparison	OTU Richness	Shannon Diversity	Community Dissimilarity
Mat vs. non-mat	$H_1 = 10.85$, $p < 0.001$	$H_1 = 16.5$, $p < 0.001$	$^*F_{1,21} = 7.01$, $p = 0.001$<br>$R^2 = 0.250$
L. gentianeus mat *vs. L. gentianeus* non-mat	$p = 0.007$	$p = 0.007$	$F_{1,9} = 20.32$, $p = 0.004$<br>$R^2 = 0.693$
L. gentianeus mat vs. L. albissimus mat	$p = 0.714$	$p = 1.000$	$F_{1,9} = 29.51$, $p = 0.003$<br>$R^2 = 0.766$
L. gentianeus mat *vs. L. albissimus* non-mat	$p = 0.007$	$p = 0.007$	$F_{1,9} = 8.90$, $p = 0.003$<br>$R^2 = 0.497$
L. gentianeus non-mat *vs. L. albissimus* mat	$p = 0.007$	$p = 0.007$	$F_{1,9} = 14.29$, $p = 0.002$<br>$R^2 = 0.588$
L. gentianeus non-mat *vs. L. albissimus* non-mat	p = 0.714	$p = 0.982$	$F_{1,9} = 4.94$, $p = 0.003$<br>$R^2 = 0.330$
L. albissimus mat *vs. L. albisimus* non-mat	$p = 0.007$	$p = 0.007$	$F_{1,9} = 6.93$, $p = 0.002$<br>$R^2 = 0.409$

Supplementary Table 1. Fungal richness, diversity, and community dissimilarity of mat vs. non-mat samples (*blocked by host species), and pairwise comparisons among these samples. The Kruskal-Wallis test was used to determine differences in OTU richness and Shannon diversity; the pairwise Wilcoxon test was used to determine differences among the pairs with Benjamini-Hochberg correction. Bray-Curtis distance was used to compare community dissimilarity. F-statistics and R^2 are reported when available.
